# Dorsal Root Ganglion Stimulation as Treatment for Complex Regional Pain Syndrome of the Foot Refractory to Spinal Cord Stimulation: A Case Report

**DOI:** 10.7759/cureus.12753

**Published:** 2021-01-17

**Authors:** Kailash Pendem, Navdeep Jassal

**Affiliations:** 1 Physical Medicine and Rehabilitation, University of Central Florida College of Medicine, Orlando, USA; 2 Pain Management, University of South Florida, Tampa, USA

**Keywords:** complex regional pain syndrome, dorsal root ganglion, spinal cord stimulation, salvage

## Abstract

The ACCURATE study demonstrated non-inferiority and superiority of dorsal root ganglion stimulation (DRGS) over traditional spinal cord stimulation (t-SCS) in patients with complex regional pain syndrome (CRPS). Yet, its efficacy in patients who had previously failed SCS therapy is unknown. A mid-60-year-old-male patient with right foot CRPS previously implanted with SCS system experienced relapse in pain. The patient was offered DRGS at right L5/S1 with 90% pain reduction. The patient subsequently proceeded to implant with continued pain relief. This case report demonstrates the effectiveness of DRGS in a patient who previously failed SCS. DRGS can be a viable salvage treatment option in the face of tolerance or loss of efficacy with SCS.

## Introduction

Complex regional pain syndrome (CRPS) was previously called reflex sympathetic dystrophy (RSD), “causalgia,” or reflex neurovascular dystrophy (RND) [1]. The Budapest consensus criteria for the clinical diagnosis of CRPS include continuing pain disproportionate to any inciting event and the patient must report at least one symptom in three of the following four categories: sensory, vasomotor, sudomotor/edema, and motor/trophic [2].

The consensus definition of CRPS describes an array of painful conditions that are characterized by regional pain that is disproportionate in time or degree to the usual course of any known trauma or lesion and shows variable progression over time [2]. There are two subtypes of CRPS that have been recognized. Type I corresponds to patients with CRPS without evidence of peripheral nerve injury. Type II describes patients with peripheral nerve injury [2]. The incidence of CRPS in population-based studies is between 5 and 26 per 100,000 per year [3].

Spinal cord stimulation (SCS), a procedure in which electrodes are placed into the dorsal epidural space, is an available treatment option for neuropathic pain conditions such as failed back surgery syndrome and CRPS. In a randomized study involving 24 patients with CRPS, SCS plus physical therapy (PT) reduced pain and improved health-related quality of life more than PT alone for up to two years. Yet, no difference in pain was perceived during three to five years following implantation [4].

Advances in neuromodulation technology have led to the development and implementation of dorsal root ganglia stimulation (DRGS). An increasing number of pain centers are using DRGS as either first-line neuromodulation or in cases where SCS has failed. The ACCURATE study was a prospective, randomized, multi-center, controlled clinical trial that demonstrated non-inferiority and superiority of DRGS over traditional SCS (t-SCS) at three months, with results sustained at 12 months. Data from the study suggests that DRGS could be used in patients suffering from chronic intractable pain conditions that are refractory to t-SCS [5].

Yang and Hunter reported a case series of two CRPS patients who failed t-SCS and were offered DRGS as salvage therapy [6]. The first patient reported 90% pain reduction during the DRG trial. The second patient underwent a surgical revision whereby a DRG-SCS system was added to the existing t-SCS to create a hybrid system with two implantable pulse generators. The patient reported immediate improvement in pain. The authors concluded that DRG-SCS was a possible salvage option in patients who failed t-SCS in CRPS.

Although recent data support DRGS over t-SCS in patients with CRPS, there is little research on effective treatment options in patients who have failed t-SCS. In this case report, we present a patient with CRPS refractory to t-SCS who was successfully treated with DRGS.

## Case presentation

We present the case of a mid-60-year-old male who suffered a tear of the right posterior tibial tendon due to a motor vehicle accident years ago. After fixation and immobilization, the foot remained painful and swollen, while the healing of the fracture was considered to be favorable. After six months, CRPS was suspected and the patient referred to a pain medicine specialist. CRPS diagnosis was confirmed according to the following criteria: continued pain disproportionate (allodynia) to the inciting event, foot hyperesthesia, skin color (redness), stiffness, and weakness of the foot and ankle (positive Budapest criteria). The patient was offered a DRGS trial at right L5 and S1 with 90% pain reduction; however, his commercial insurance plan failed to cover the DRGS implantation, deeming it experimental. He then underwent a Boston Scientific SCS trial with >50% relief and was implanted with an Boston Scientific (Marlborough, MA, USA) SCS system (16-contact lead placed at the top of T8) in 2019. After a successful trial, the patient reported 80% pain relief with the SCS system. Unfortunately, the patient reported worsening of pain symptoms after four months. Imaging was obtained to rule out lead migration. The system was reprogrammed several times without success for pain relief, with multiple wave forms being trialed. Unfortunately, none could recreate his pain relief and functional improvement from the prior DRGS trial.

The patient presented to our clinic with right foot pain that he reported as eight out of ten on the numeric rating scale (NRS). He complained that he is easily fatigued with walking. Physical examination of the right foot was notable for allodynia and nail discoloration.

With a change in his insurance plan, he subsequently proceeded with a DRGS implantation that was approached in the same manner as described by Liem et al. [7]. The Abbott DRGS System (Abbott, TX, USA) was utilized.

DRG leads were placed at the right L5/S1 and S1 neural foramen. Adequate paresthesia mapping was achieved intraoperatively over his painful right lower extremity. Three days after the implantation, the patient reported greater than 90% improvement in CRPS pain. Furthermore, the patient was ambulating with one out of ten on the NRS.

On his six month follow-up appointment, the patient mentioned rare pain flare ups of eight to nine out of ten on the NRS and had weaned himself off opioid medication (oxycodone/acetaminophen tablets). He occasionally felt “flutter” of his posterior distal leg muscles. These issues were remedied when the DRG system was reprogrammed. His pain score has remained one out of ten most times. The device has been used 24 hours a day. The patient’s SCS system was explanted after achieving superior relief with his Abbott DRG neurostimulation system. Verbal informed consent was obtained from the patient for anonymized patient information to be published in this article.

## Discussion

Tolerance to neurostimulation is one of the greatest challenges to long-term efficacy in neuromodulation for chronic pain [8]. There is no agreed-upon treatment algorithm for CRPS once a patient stops responding to SCS for an unknown reason. Although the ACCURATE study demonstrated non-inferiority and superiority of DRGS over t-SCS, there is little research on whether it should be utilized after failure of t-SCS.

The patient presented in this case reported a decline in the ability of the SCS device in relieving pain. Hardware-associated failure of SCS (e.g., resulting from physical damage to or migration of leads) was ruled out by troubleshooting and reprogramming attempts by the manufacturer. X-ray ruled out obvious lead fracture or migration. The exact etiology of failure of SCS device in this patient is still unknown. In our patient, DRGS therapy resulted in a clinically significant result. The pain level went from an initial NRS score of eight to one to two and he regained increased ambulatory ability. Our results with DRGS therapy are promising and in line with another published case report [6].

## Conclusions

This case report demonstrates the effectiveness of DRGS in a patient who previously failed SCS. DRGS can be a viable salvage treatment option in the face of tolerance or loss of efficacy with SCS. Although a case report is related to the care of an individual patient, it can be a source of new ideas in medicine, providing treatment guidance for clinical practice and generate hypotheses. To date, our patient remains satisfied with DRGS and has not experienced any complications related to the neurostimulation device.
